# Impact of reducing the duration of fasting and no drinking on the experiences of older patients receiving painless gastroscopy: a randomized controlled trial

**DOI:** 10.7717/peerj.20929

**Published:** 2026-03-11

**Authors:** Yong Lan, Xin Sun, Tengjiang Yu, Tao He, Liang Ma, Yuexi Chen, Jiaxin Tian, Xia Jiang, Qingfeng Jiang, Wusheng Li, Weidong Chen, Shichao Li

**Affiliations:** 1 Endoscope Center, The Affiliated Traditional Chinese Medicine Hospital, Southwest Medical University, Luzhou, China; 2 Department of Anorectal, The Affiliated Traditional Chinese Medicine Hospital, Southwest Medical University, Luzhou, China; 3 The Key Laboratory of Integrated Traditional Chinese and Western Medicine for Prevention and Treatment of Digestive System Diseases of Luzhou City, Luzhou, China

**Keywords:** Older patients, Painless gastroscopy, Patient comfort, Fasting, No drinking

## Abstract

**Background:**

Before undergoing painless gastroscopy, patients are required to fast and no drinking for at least 4 h which is unpleasant for older patients.

**Objective:**

The aim of this study is to increase the preprocedural comfort of older patients by reducing the duration of fasting and no drinking before painless gastroscopy while ensuring safety.

**Methods:**

Older patients aged more than 65 years who underwent painless gastroscopy were randomly allocated to the study group (*n* = 452) and the control group (*n* = 452). Those in the study group consumed 250 ml of opaque liquid 4 h prior to painless gastroscopy and 250 ml of water 2 h before the procedure. Those in the control group consumed the same food and water as those in the study group for 6–8 and 4 h, respectively. The primary outcome was patient comfort before the examination. The secondary outcomes included safety, gastroscopy effect and satisfaction in each group. All outcomes were analyzed using both intention-to-treat (ITT) and per-protocol (PP) approaches.

**Results:**

In the study group, the incidence of thirst, hunger, dizziness, and fatigue before gastroscopy was significantly lower than that in the control group (33.4% *vs*. 42.7%, 20.1% *vs*. 28.1%, 3.1% *vs*. 7.5% and 4.9% *vs*. 11.7%, respectively; all *P* < 0.01). However, in the gastroscopic assessments of both groups, only a small fraction of the patients exhibited gastric reflux, gastric fluid or food retention, aspiration pneumonia (2.0% *vs*. 0.9%, 4.4% *vs*. 3.1%, 1.5% *vs*. 1.1%, 0.4% *vs*. 0.2%, respectively; all *P* > 0.05).Additionally, there was no difference in visual field clarity between the two groups (1.00 (1.00, 2.00) *vs*. 1.00 (1.00, 2.00), *P* > 0.05), but the satisfaction of the study group was significantly greater than that of the control group (97.00 (96.00, 99.00) *vs*. 93.00 (92.00, 95.00), *P* < 0.01).

**Conclusion:**

In this random trial of older patients undergoing painless gastroscopy, ingestion of 250 ml opaque liquid 4 h before, followed by 250 ml water 2 h before, improved pre-procedure comfort and patient satisfaction compared with conventional fasting. We observed no statistically significant increase in clinically identified gastric reflux, aspiration, or impaired endoscopic field clarity; however, the incidence of such adverse events was low, and the trial was not powered to exclude small increases in rare but serious complications. Larger studies would be required to definitively establish safety equivalence.

## Introduction

Enhancements in contemporary medical practices have notably prolonged human lifespans. According to the Global Burden of Disease (GBD) study, the average life expectancy at birth globally and in China increased to 73.8 years and 80.1 years, respectively, in 2023 (Collaborators, 2025). China already has the world’s largest number of older people, and the number is growing. By 2050, 395 million people will be aged 65 and older in China (Chen et al., 2022). Ideally, prolonged lifespans correlate with better bodily health. However, since 2000, this trend has reversed, with longer lifespans leading to declining health (Zhao et al., 2019). With advancing age, organ function decreases, immunity weakens, and the occurrence of various illnesses, such as digestive disorders, increases. Gastroscopy is a crucial diagnostic and treatment tool for upper gastrointestinal ailments, and with the advent of comfort medicine, painless gastroscopy has become the preferred method for treating older patients. Fasting before surgery is crucial for painless gastroscopy, and rigorous fasting can lower the chances of reflux, fatal aspiration pneumonia, and other grave complications (Andersson, Schmitz & Frykholm, 2018; Thomas et al., 2018). Nevertheless, as enhanced recovery after surgery (ERAS) becomes more prominent (Brown & Xhaja, 2018), research has indicated that prolonged fasting before surgery may result in malnutrition, leading to extended hospitalizations and increased rates of readmission and death (De Jonghe et al., 2016). Surgeons and anesthetists have cast doubt on the conventional practices of fasting and refraining from consuming food and beverages, following extended clinical experience (Bloc et al., 2023).

Painless gastroscopy is the most used method for the diagnosis and treatment of upper digestive tract diseases (Zheng et al., 2023). Painless gastroscopy requires the use of one or more sedatives/anesthetics to guarantee that patients safely undergo endoscopic examination and treatment devoid of pain and in proper light sleep (Ladas et al., 2010). Before painless gastroscopy, patients are routinely advised to fast for 6–8 h and refrain from drinking water for 4 h to prevent serious problems, such as aspiration pneumonia (Zhu et al., 2021). However, the fasting duration for most patients who undergo painless gastroscopy significantly exceeds this period in real clinical procedures, sometimes reaching 16 h (Koeppe et al., 2013; Li et al., 2021). Extended fasting periods may lead to mental and physical strain on patients (Lua et al., 2022). Individuals with this condition are more susceptible to negative effects, such as thirst, hunger, irritability, anxiety, electrolyte imbalances, and metabolic irregularities, with a heightened risk of postanesthetic reflux (Ducey & Nikoo, 2018; Napolitano et al., 2021), which is particularly evident in this specific older patient demographic (Blommers et al., 2011).

With the decline in organ function, older patients frequently face a mix of age-related chronic illnesses and prolonged oral intake of diverse therapeutic medications (Rieckert et al., 2018), significantly increasing their anesthesia risk (Lee, Marhalik-Helms & Penzi, 2023). Furthermore, extended periods of fasting frequently result in symptoms such as dizziness, headache, nausea, and various other issues during the preparatory phase (Leslie, Tay & Neo, 2006), potentially leading to complications such as low blood pressure and cardiac arrhythmia during surgery. Prolonged abstinence from food and beverage consumption, leading to these negative effects, significantly diminishes the mental and physical well-being of older patients, thus impacting their health care experience (Kurrek, Barnett & Minville, 2014). Previously, the American Society of Anesthesiologists (ASA) recommended that adult patients who undergo elective procedures may consume up to 400 ml of clear liquids until 2 h before surgery (Joshi et al., 2023). Practice guidelines from the Indian Society of Anaesthesiologists recommend that adults may consume <450 ml of clear liquids 2 h prior to anesthesia and an appropriate amount of opaque liquids 4 h prior to anesthesia (Dongare et al., 2020). However, few studies have investigated on the specific measures and safety of this recommendation for older patients undergoing painless gastroscopy. Consequently, the aim of this study was to explore how reducing the duration of fasting and no drinking before painless gastroscopy affects the experience and safety of older patients.

## Materials and Methods

### Study design and participants

This prospective randomized controlled trial was conducted at the Affiliated Hospital of Traditional Chinese Medicine of Southwest Medical University between June 25, 2023 and December 31, 2023. This study was approved by the Ethics Committee of the Affiliated Hospital of Traditional Chinese Medicine of Southwest Medical University (BY2023010) and was registered in the Chinese Clinical Trial Registry (https://www.chictr.org.cn/, ChiCTR2300072760). All patients signed written informed consent before the procedure. All procedures of this study adhered to CONSORT guidelines.

Patients were eligible if they were ≥65 years of age, were classified as ASA class I or II, and presented no significant drug contraindications. The exclusion criteria included critical cardiovascular and respiratory conditions or liver dysfunction (Child‒Pugh class C or above), sudden upper gastrointestinal hemorrhage accompanied by shock, blockage in the gastrointestinal tract, or allergic reactions to sedatives/anesthetics, along with other grave anesthesia hazards.

### Procedure

The patients in this study were outpatients and inpatients at the Affiliated Hospital of Traditional Chinese Medicine of Southwest Medical University. Eligible patients were randomly separated into two groups, a study group and a control group. Researcher Wusheng Li generated random sequences through SPSS 23.0 software (IBM Corp., Armonk, NY, USA), and put them in opaque envelopes in sequence. The appointment nurse opened the envelope at least one day before the gastroscopy appointment and informed eligible patients of the plan of no drinking and fasting before painless gastroscope. Participants in the study group underwent a 4 h fasting period (consuming 250 ml of milk or rice porridge 4 h before the examination) and a 2 h no-drinking period (consuming 250 ml of water 2 h before the examination), whereas the control group followed routine fasting for 6–8 h and not drinking for 4 h. Pure milk was purchased from Inner Mongolia Mengniu Dairy Group Co., Ltd. (Hohhot, Inner Mongolia) and the macronutrients per 100 ml were as follows: protein, 3.2 g; fat, 4.0 g; and carbohydrate, 4.8 g. The rice porridge was prepared daily by the hospital cook for older patients undergoing gastroscopy. To prepare the porridge, 50 g rice with 1.0 L boiled water was cooked by simmering over a low flame for 1 h. Macronutrients per 100 g rice porridge: carbohydrate: 6–10 g, protein: 0.5–1 g. Fat: <0.3 g.

To enhance patient cooperation, researchers explained the necessity of fasting from food and drinks the day before the procedure. They documented the duration and types of fasting (solid and liquid) on the day of the test. Prior to anesthesia, participants were queried about symptoms such as thirst, hunger, dizziness, and fatigue. During the examination, endoscopists evaluated potential complications, including gastric reflux, gastric food retention, gastric fluid retention, and aspiration pneumonia, and assessed the clarity of the gastroscope field. After painless gastroscopy, patients satisfaction with the entire process was evaluated by a questionnaire survey. Investigators who collected pre-operative comfort data were unaware of patient group assignment at the time of assessment. All the researchers who participated in the data collection of the protocol remained blinded to group allocation until study completion.

The anesthesia scheme for painless gastroscopy involved a combination of intravenous propofol (2–3 mg/kg) and sufentanil (5–8 µg/kg). Before painless gastroscopy was performed, evaluating the physical condition of patients with respect to the ASA was necessary to classify patients under anesthesia. Painless gastroscopy was performed using a Fujinon BL-7000 (Fujinon, Japan). All the operating endoscopists were trained in the study protocol and used the same evaluation form to evaluate the patient’s stomach conditions without knowing the fasting state of the patient.

### Outcomes

The primary outcome was patient comfort before the examination: the incidence of thirst, hunger, dizziness and fatigue, which was evaluated by the blinded research nurse before anesthesia. Thirst, dizziness, and fatigue were defined according to the Common Terminology Criteria for Adverse Events (CTCAE) version 5.0 criteria (National Institutes of Health & National Cancer Institute, 2017). Hunger was defined using the physiological hunger subscale of the General Food Cravings Questionnaire-State (G-FCQ-S), which assesses craving for food related to physiological needs (Nijs, Franken & Muris, 2007). Thirst incidence was prespecified as the primary endpoint; all other indices–including the incidence of hunger, dizziness, and fatigue–were designated exploratory endpoints. Patient comfort was the primary outcome approved by the Ethics Committee (May 12, 2023), but the primary outcome of clinical registration is safety (June 12, 2023). We reported patient comfort as the primary outcome and followed the study protocol approved by the Ethics Committee.

One of the secondary outcomes was safety during painless gastroscopy: after the gastroscopy examination was completed, the examining physician evaluated safety during the gastroscopy examination, including whether there was any gastric reflux, gastric food or fluid retention, and whether there was any aspiration pneumonia. Gastric reflux was defined as the reflux of gastric fluid or contents to the esophagus or even the oral cavity during the insertion or manipulation of the gastroscope (Antono & Dotson, 2025). Gastric fluid retention was characterized by the accumulation of gastric fluid that was not promptly emptied and exceeded 200 ml during gastroscopy. Gastric food retention was indicated by the presence of a large amount of food residue, exceeding 200 ml, in the stomach during gastroscopy. Gastric fluid volume was quantified as the total volume aspirated endoscopically into the collection reservoir. For assessment of residual solid gastric content, the cross-sectional area (CSA) of the gastric antrum was measured by ultrasonography and the volume was estimated from this single-plane CSA value (Ohashi et al., 2018). Aspiration pneumonia was diagnosed when patients exhibited new-onset lung consolidation on chest radiography or CT scans, along with respiratory infection symptoms such as fever and oxygen desaturation. During gastroscopy, aspiration pneumonia often manifests as cough, dyspnoe and decreased oxygen saturation (Matsumi et al., 2017).

Other secondary outcomes included clarity of the visual field: This information was filled out and evaluated by the examining physician on the basis of the mucous membrane appearance during the examination, and visual clarity was judged and assessed by a 10-point Likert scale (Jebb, Ng & Tay, 2021). The degree of restriction of mucosal observation under gastroscopy was defined as no restriction (0 points), mild restriction (1–3 points), moderate restriction (4–6 points), severe restriction (7–9 points), or mucosa not assessable (10 points). Immediately after gastroscopy insertion, a fixed endoscopist scored the participants blindly.

Another secondary outcome was overall patient satisfaction, which was scored on a scale of 0–100 points. In addition, the mean arterial pressure (MAP, (systolic blood pressure + 2 × diastolic blood pressure)/3), heart rate (HR), and peripheral oxygen saturation (SpO2) were also measured for both groups at different time points, specifically at the onset of anesthesia, at the end of anesthesia, and before the patients left the recovery room.

### Statistical analysis

Sample size calculation was conducted using PASS version 11.0 (NCSS LLC, Kaysville, Utah, USA). On the basis of the results of a previous study (*n* = 44 per group) (Greenfield et al., 1996), the incidence of thirst was 70.45% (31/44) in the starvers group and the incidence of thirst in older patients in our study group was estimated to be 60%. Consequently, a minimum sample size of 870 cases (435 cases in each group) was determined, assuming a two-tailed α = 0.05, 90% power of detection, and a 1:1 allocation ratio. Considering a potential dropout rate of 4%, 452 patients were included in each group.

SPSS 23.0 (IBM Corp., Armonk, NY, USA) statistical software was used for statistical analysis. Descriptive measurement data are described as the means ± standard deviations or medians (interquartile range) depending on the validity of the assumption of normality. Group comparisons were carried out using independent t tests or Mann‒Whitney U tests, as appropriate. Enumeration data are expressed as numbers and percentages. Chi-square tests or Fisher’s exact tests were employed for between-group comparisons of enumeration data. All primary and secondary outcomes were analyzed according to both the intention-to-treat (ITT) and per-protocol (PP) principles. In the ITT set, missing data were addressed exclusively for the primary outcome (patient comfort) by imputing the worst observation carried forward (WOCF); no imputation was applied to any secondary outcomes. Statistical significance was set at *P* < 0.05 for all analyses.

## Results

### Baseline characteristics

**Figure 1:**
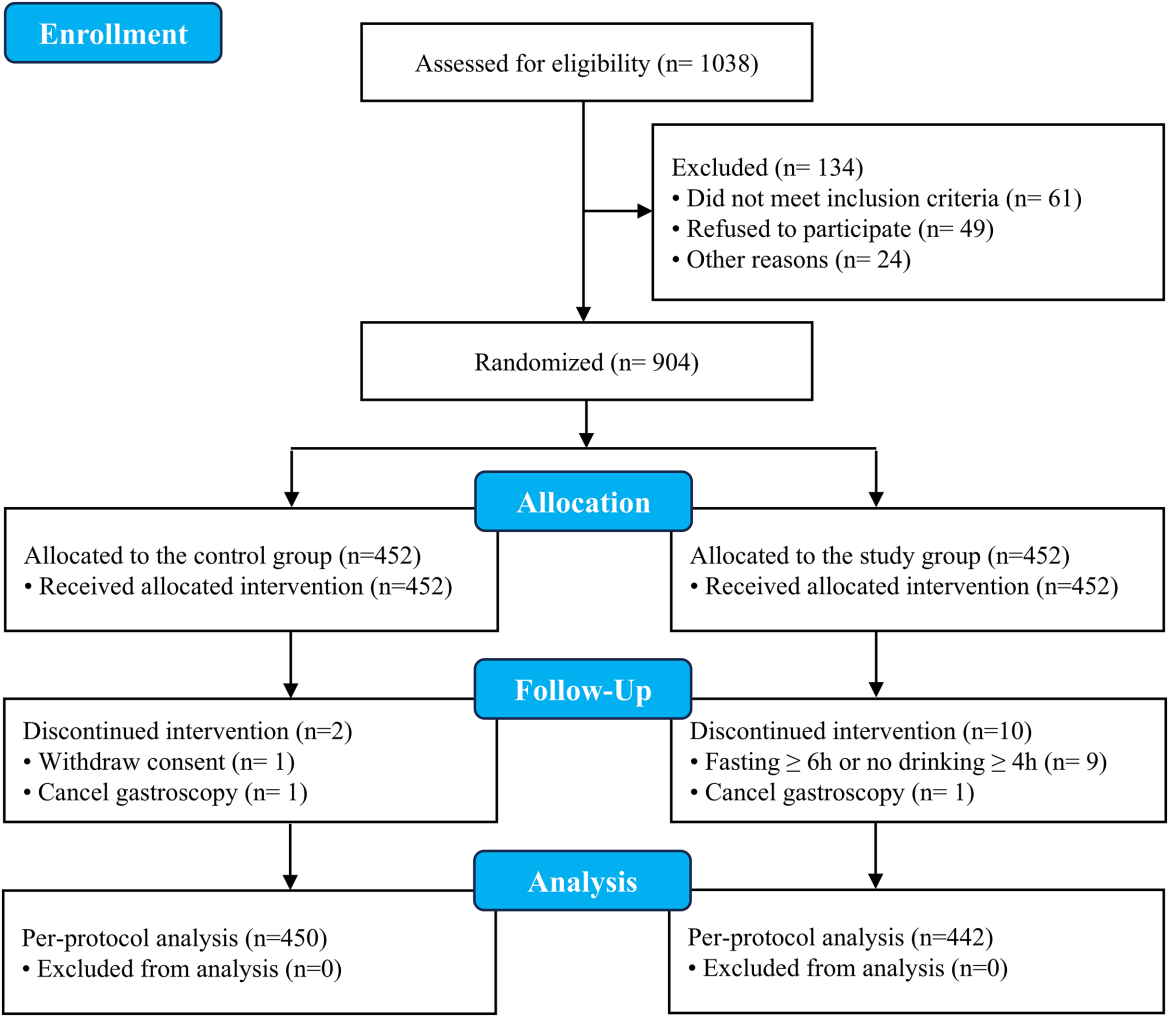
CONSORT diagram.

As shown in Fig. 1, a total of 1038 patients were assessed for eligibility; among them, 904 patients who fulfilled the inclusion criteria and provided informed consent were enrolled in the study. These patients were randomly and equally assigned to either the control group or the study group (452 patients in each group). Actual fasting time was significantly shorter in the study group than in the control group (median 4.5 h [interquartile range (IQR) 4–5 h] *vs*. 6 h (IQR 6–6.5 h); range 4–8 h *vs*. 6–8 h). Similarly, no drinking time was reduced in the study group {median 2 h (IQR 2–2.6 h) *vs*. 4 h (IQR 4–4.5 h); range 2–4.9 h *vs*. 4–5.8 h}. Compliance with fasting protocols was high in both groups, with 442 (97.8%) patients in the study group and 450 (99.6%) in the control group adhering to the prescribed guidelines. Twelve patients were excluded because the prescribed fasting and water ban time was exceeded, the gastroscope was cancelled, or consent was withdrawn. The final PP analysis included 442 patients in the study group and 450 in the control group. With respect to the ITT analysis baseline characteristics, there were no significant differences in sex, age, height, weight, BMI, ASA grade, history of hypertension or history of type 2 diabetes between the two intervention groups. All baseline standardized mean differences (SMDs) (Hedges’ g) were < 0.1, indicating negligible imbalance between groups. Moreover, there was no significant difference in gastroscopy findings between the two groups (Table 1).

**Table 1:** General information and gastroscopy fndings of patients in both groups.

	Control group (*n* = 452)	Study group (*n* = 452)	SMDs (95% CI)	*P*
Gender			–	0.548
Male	212 (46.9%)	203 (44.9%)		
Female	240 (53.1%)	249 (55.1%)		
Age (years)	71.33 ± 5.08	71.50 ± 5.07	−0.03 [−0.15 to 0.08]	0.619
Height (cm)	162.08 ± 7.42	162.69 ± 7.52	−0.08 [−0.20 to 0.03]	0.223
Weight (kg)	61.47 ± 8.41	62.09 ± 8.61	−0.07 [−0.19 to 0.04]	0.276
BMI (kg/m^2^)	23.19 (21.21, 25.39)	23.05 (21.10, 26.03)		0.820
ASA grade			–	0.737
Grade I	195 (43.1%)	200 (44.2%)		
Grade II	257 (56.9%)	252 (55.8%)		
Hypertension	267 (60.6%)	281 (62.2%)	–	0.341
Type 2 diabetes	105 (23.8%)	110 (24.3%)	–	0.696
Gastroscopy findings				
Gastric polyp	125 (27.7%)	127 (28.1%)	–	0.882
Chronic atrophic gastritis	93 (20.4%)	112 (24.8%)	–	0.131
Chronic non-atrophic gastritis	53 (11.7%)	59 (13.1%)	–	0.545
Gastric ulcer	34 (7.5%)	33 (7.3%)	–	0.899
Duodenal ulcer	26 (5.8%)	17 (3.8%)	–	0.160
Gastric elevated lesion	16 (3.5%)	15 (3.3%)	–	0.855
Esophagitis	15 (3.3%)	15 (3.3%)	–	1.000
Hiatal hernia	12 (2.7%)	8 (1.8%)	–	0.366
Esophageal and gastric varices	12 (2.7%)	8 (1.8%)	–	0.366
Gastric superficial lesion	11 (2.4%)	6 (1.3%)	–	0.221
Gastric submucosal lesion	8 (1.8%)	12 (2.7%)	–	0.366
Esophageal cancer	8 (1.8%)	7 (1.5%)	–	0.795
Gastric cancer	5 (1.1%)	6 (1.3%)	–	1.000
Others	33 (7.3%)	26 (5.8%)	–	0.346

### Outcomes

Table 2 presents the primary outcome of the two groups in the ITT and PP population. In the ITT analysis, the findings revealed that the incidence of discomfort such as thirst (33.4% *vs*. 42.7%, relative risk (RR) = 0.78 (95% CI [0.66–0.93]), absolute risk reduction (ARR) = 9.29% (95% CI [2.99–15.58%]), number needed to treat (NNT) = 11 (95% CI [6–33]), *P* = 0.004)), hunger (20.1% *vs*. 28.1%, RR = 0.72 (95% CI [0.57–0.90]), ARR = 7.97% (95% CI [2.31– 13.63%]), NNT = 13 (95% CI [7–43]), *P* = 0.004), dizziness (3.1% *vs*. 7.5%, RR = 0.41 (95% CI [0.22–0.76]), ARR = 4.42%(95% CI [1.83–7.01%]), NNT = 23 (95% CI [14–55]), *P* = 0.002) and fatigue (4.9% *vs*. 11.7%, RR = 0.41 (95% CI [0.26–0.67]), ARR = 6.86% (95% CI [3.46–10.26%]), NNT = 15 (95% CI, 10, 29), *P* < 0.001), was lower in the study group than in the control group. After adjusting for ASA grade, type 2 diabetes history, and BMI using multivariable logistic regression, the study group demonstrated significantly reduced risks for all four symptoms compared to the control group: thirst: adjusted odds ratio (OR) = 0.67 (95% CI [0.52–0.87]), ARR = 8.92% (95% CI [3.14–14.70%]); hunger: adjusted OR = 0.65 (95% CI [0.48–0.88]), ARR = 7.75% (95% CI [2.18– 13.32%]); dizziness: adjusted OR = 0.38 (95% CI [0.20–0.73]), ARR = 4.17% (95% CI [1.57–6.77%]); fatigue: adjusted OR = 0.39 (95% CI [0.23–0.66]), ARR = 6.50% (95% CI [3.10–9.90%]). All adjusted ORs were statistically significant, with CIs excluding 1.0. These results were consistent in the PP populations.

**Table 2:** Comfort level between the two groups in the ITT and PP population.

	Control group	Study group	RR (95% CI)	ARR (%) (95% CI)	NNT (95% CI)	Adjusted OR (95% CI)	Adjusted ARR (%) (95% CI)	*P*
ITT population (*n* = 452:452)								
Thirsty	193 (42.7%)	151 (33.4%)	0.78 [0.66–0.92]	9.29 [2.99–15.58]	11 [6–33]	0.67 [0.52–0.87]	8.92 [3.14–14.70]	0.004
Hunger	127 (28.1%)	91 (20.1%)	0.72 [0.58–0.89]	7.97 [2.31–13.63]	13 [7–43]	0.65 [0.48–0.88]	7.75 [2.18–13.32]	0.004
Dizziness	34 (7.5%)	14 (3.1%)	0.41 [0.22–0.77]	4.42 [1.83–7.01]	23 [14–55]	0.38 [0.20–0.73]	4.17 [1.57–6.77]	0.002
Fatigue	53 (11.7%)	22 (4.9%)	0.42 [0.26–0.67]	6.86 [3.46–10.26]	15 [10–29]	0.39 [0.23–0.66]	6.50 [3.10–9.90]	<0.001
PP population (*n* = 450:442)								
Thirsty	191 (42.4%)	147 (33.3%)	0.78 [0.66–0.93]	9.19 [2.85–15.52]	11 [6–35]	0.67 [0.51–0.87]	8.82 [3.04–14.60]	0.005
Hunger	126 (28.0%)	89 (20.1%)	0.72 [0.58–0.90]	7.86 [2.28–13.45]	13 [7–44]	0.64 [0.47–0.88]	7.63 [2.08–13.18]	0.006
Dizziness	33 (7.3%)	12 (2.7%)	0.37 [0.19–0.72]	4.62 [1.77–7.46]	22 [13–56]	0.34 [0.17–0.69]	4.34 [1.60–7.08]	0.002
Fatigue	52 (11.6%)	18 (4.1%)	0.35 [0.21–0.60]	7.48 [4.00–10.96]	13 [9–25]	0.32 [0.18–0.57]	6.50 [3.10–9.90]	<0.001

**Note:**

RR, relative risk; OR, odds ratio; ARR, absolute risk reduction; NNT, number needed to treat; CI, confidence interval.

To assess the robustness of the treatment estimates, we conducted two sensitivity analyses. Firstly, the PP analysis yielded effect directions identical to those of the ITT analysis for every outcome, and all comparisons remained statistically significant. For the thirst and hunger, the RR estimates were numerically identical (RR 0.78 and 0.72, respectively). For dizziness and fatigue, the PP analysis indicated larger effect sizes (RR 0.37 and 0.35, respectively), consistent with the expected impact of excluding participants with prolonged fasting. Secondly, protocol-deviation rates were higher in the study group (2.2%) than in the control group (0.4%). The concordance between ITT and PP results indicates that, despite these deviations, the principal conclusion that the intervention significantly reduces the incidence of discomfort, is robust to the choice of analytical population. The true treatment effect most likely lies between the conservative ITT estimate and the PP estimate.

The secondary outcomes were safety results during painless gastroscopy, visual field clarity and patient satisfaction with painless gastroscopy. Table 3 shows the safety results during painless gastroscopy of the two groups in the ITT and PP population. In the ITT analysis, the incidence of gastric reflux {2.0% *vs*. 0.9%, RR = 2.25 (95% CI [0.69–7.34]), absolute risk increases (ARI) = 1.11% (95% CI [−0.34% to 2.56%]), number needed to harm (NNH) = 90 (95% CI [39, ∞, NNT 294]), Minimum detectable RR at 80% efficacy (MDRR₈₀) = 3.16, *P* = 0.269)}, gastric food retention {1.5% *vs*. 1.1%, RR = 1.40 (95% CI [0.46– 4.28]), ARI = 0.44% (95% CI [−0.73% to 1.61%]), NNH = 227 (95% CI [62, ∞, NNT 137]), MDRR₈₀ = 2.48, *P* = 0.773)}, aspiration pneumonia {0.4% *vs*. 0.2%, RR = 2.00 (95% CI [0.18–22.00]), ARI = 0.22% (95% CI [−0.41% to 0.85%]), NNH = 455 (118, ∞, NNT 244), MDRR₈₀ = 6.17, *P* = 1.000)}, and gastric fluid retention {4.4% *vs*. 3.1%, RR = 1.43 (95% CI [0.74–2.76]), ARI = 1.33% (95% CI [−0.80% to 3.46%]), NNH = 75 (95% CI [29, ∞, NNT 125]), MDRR₈₀ = 1.88, *P* = 0.294)} did not significantly differ between the study group and control group (all *P* > 0.05). These results were consistent in the PP populations. However, the number of events was low and our study was not powered to exclude small increases in rare events.

**Table 3:** Safety between the two groups in the ITT and PP population.

	Control group	Study group	RR (95% CI)	ARI (%) (95% CI)	NNH (95% CI)		MDRR_80_		*P*	
ITT population (*n* = 452:452)										
Gastric reflux	4 (0.9%)	9 (2.0%)	2.25 [0.69–7.34]	1.11 [−0.34 to 2.56]	90 [39, ∞, NNT 294]		3.16		0.269	
Gastric food retention	5 (1.1%)	7 (1.5%)	1.40 [0.46–4.28]	0.44 [−0.73 to 1.61]	227 [62, ∞, NNT 137]		2.48		0.773	
Aspiration pneumonia	1 (0.2%)	2 (0.4%)	2.00 [0.18–22.00]	0.22 [−0.41 to 0.85]	455 [118, ∞, NNT 244]		6.17		1.000	
Gastric fluid retention	14 (3.1%)	20 (4.4%)	1.43 [0.74–2.76]	1.33 [−0.80 to 3.46]	75 [29, ∞, NNT 125]		1.88		0.294	
PP population (*n* = 450:442)										
Gastric reflux	4 (0.9%)	9 (2.0%)	2.29 [0.70–7.50]	1.15 [−0.32 to 2.62]	87 [38, ∞, NNT 313]		3.16		0.173	
Gastric food retention	5 (1.1%)	7 (1.6%)	1.42 [0.46–4.38]	0.47 [−0.70 to 1.64]	213 [61, ∞, NNT 143]		2.49		0.575	
Aspiration pneumonia	1 (0.2%)	2 (0.5%)	2.04 [0.19–22.30]	0.23 [−0.40 to 0.86]	435 [116, ∞, NNT 250]		6.26		0.621	
Gastric fluid retention	13 (2.9%)	20 (4.5%)	1.57 [0.79–3.09]	1.63 [−0.45 to 3.71]	61 [27, ∞, NNT 222]		1.98		0.196	

**Note:**

ARI, absolute risk increases; NNH, number needed to harm; MDRR_80_, Minimum detectable RR at 80% efficacy.

Table 4 presents the visual field clarity and patient satisfaction with painless gastroscopy between the two groups. In the ITT and PP analysis, the difference in visual field clarity between the two groups was not statistically significant (*P* = 0.397, 0.298, respectively). Notably, comparisons of the overall satisfaction of the two groups of patients revealed that the study group had significantly greater satisfaction than the control group did (all *P* < 0.001).

**Table 4:** Field of view clarity score and patient satisfaction score between the two groups.

	ITT analysis				PP analysis		
	Control group (*n* = 452)	Study group (*n* = 452)	*P*		Control group (*n* = 450)	Study group (*n* = 442)	*P*
Field of view clarity score	1.00 (1.00, 2.00)	1.00 (1.00, 2.00)	0.397		1.00 (1.00, 2.00)	1.00 (1.00, 2.00)	0.298
Patient satisfaction score	93.00 (92.00, 95.00)	97.00 (96.00, 99.00)	<0.001		93.00 (92.00, 95.00)	98.00 (96.00, 99.00)	<0.001

Table 5 shows the comparisons of MAP, HR and SpO_2_ between the two groups at different time points. The ITT and PP analysis results revealed that there was no significant difference in the MAP, HR or SpO2 between the two groups at different time points (all *P *> 0.05).

**Table 5:** Comparison of MAP, HR and SpO2 between the two groups at different time points.

	ITT analysis					PP analysis			
	Control group (*n* = 452)	Study group (*n* = 452)	MD (95% CI)		*P*	Control group (*n* = 450)	Study group (*n* = 442)	MD (95% CI)	*P*
MAP (mmHg)									
T1	93.13 ± 9.55	93.86 ± 9.36	0.73 [−0.51 to 1.97]		0.247	93.15 ± 9.55	93.98 ± 9.36	0.84 [−0.40 to 2.08]	0.185
T2	92.13 ± 9.16	92.65 ± 8.85	0.55 [−0.63 to 1.72]		0.362	92.13 ± 9.16	92.78 ± 8.84	0.66 [−0.53 to 184]	0.276
T3	92.23 ± 9.40	92.80 ± 9.00	0.57 [−0.63 to 1.77]		0.349	92.24 ± 9.24	92.92 ± 9.00	0.68 [−0.53 to 1.88]	0.269
HR (bpm)									
T1	77.44 ± 10.33	76.96 ± 10.77	−0.48 [−1.86 to 0.90]		0.498	77.44 ± 10.34	77.07 ± 10.83	−0.38 [−1.77 to 1.01]	0.593
T2	75.93 ± 9.39	75.49 ± 9.83	−0.44 [−1.69 to 0.82]		0.495	75.94 ± 9.40	75.61 ± 9.88	−0.33 [−1.60 to 0.93]	0.605
T3	76.29 ± 9.42	75.78 ± 9.88	−0.51 [−1.77 to 0.75]		0.430	76.29 ± 9.43	75.88 ± 9.94	−0.40 [−1.67 to 0.87]	0.536
SpO_2_ (%)									
T1	96.27 ± 2.92	96.36 ± 2.83	0.19 [−0.28 to 0.47]		0.626	96.26 ± 2.92	96.38 ± 2.83	0.12 [−0.26 to 0.49]	0.548
T2	98.00 (95.00, 98.00)	98.00 (95.00, 98.00)	–		0.332	98.00 (95.00, 98.00)	98.00 (95.00, 98.00)	–	0.310
T3	96.36 ± 2.61	96.53 ± 2.53	0.17 [−0.16 to 0.51]		0.316	96.35 ± 2.61	96.52 ± 2.55	0.17 [−0.17 to 0.51]	0.337

**Note:**

T1: At the onset of anesthesia; T2: At the end of anesthesia; T3: Before leaving the resuscitation room. MD, Mean Difference.

## Discussion

While ERAS protocols have successfully implemented abbreviated fasting with carbohydrate-rich drinks up to 2 h before surgery in adult populations, evidence specific to older patients undergoing painless gastroscopy remains limited (Joshi et al., 2023). Older patients represent a kind of clinical population with unique physiology: age-related delays in gastric emptying (Salive, 2013), a higher prevalence of comorbidities, and polypharmacy (Patejdl, 2024) may alter their responses to preoperative fasting regimens and increase their risk of discomfort and dehydration. Therefore, directly extrapolating existing ERAS fasting guidelines to older adults undergoing gastroscopy may not be appropriate. This randomized trial demonstrates that the ingestion of 250 ml of opaque liquid food 4 h prior to painless gastroscopy, followed by the consumption of 250 ml of water 2 h before the procedure, significantly increased the comfort and satisfaction of older patients. Importantly, this comfort benefit was achieved without a significant increase in clinically relevant adverse events; we observed no significant rise in gastric reflux, gastric food or fluid retention, aspiration pneumonia, or impairment in endoscopic visual field clarity. Consequently, this approach may represent a feasible and patient-centered alternative for patients aged ≥65 years with ASA grade I–II undergoing elective painless gastroscopy.

One study reported that it was safe for elective patients to drink 330 ml of water 2 h before general gastroscopy which alleviated symptoms of thirst (Greenfield et al., 1996). Lua et al.’s (2022) study revealed that a commercial carbohydrate-rich whey protein beverage taken orally 2 h before gastroscopy under local anesthesia resulted in higher gastric residue and better well-being and safety than oral intake of water. A study on painless gastroscopy revealed that, compared with patients who underwent conventional fasting, patients who underwent painless gastroscopy after a 2 h fast experienced greater comfort and reduced anxiety, general discomfort, hunger, and weakness, with no safety concerns (Koeppe et al., 2013). Another study on painless gastrointestinal endoscopy for polyp treatment demonstrated that shortening the preoperative fasting time for both solids and liquids could stabilize blood glucose, insulin, and electrolyte levels before and after the procedure and alleviate preoperative thirst and hunger (Li et al., 2021). These findings align with our observations that the study group of patients experienced notably fewer instances of discomfort including thirst, hunger, dizziness, and fatigue before the examination than did the control group did. In contrast, our research focused on the influence of shortening the fasting and no drinking times of older patients before painless gastroscopy on their experience and safety.

Older patients face a heightened aspiration risk owing to diminished sensitivity in the pharynx and weakened gastric emptying ability (Kao et al., 1994; Razavi, Gross & Katz, 2014). The primary objective of fasting prior to painless gastroscopy is to mitigate the risk of aspiration during anesthesia. However, recent findings have indicated that aspiration is only likely to occur when the gastric content volume (GCV) exceeds 200 ml (Li et al., 2021). Consequently, a preoperative preparation protocol aimed at reducing the duration of fasting and not drinking has been proposed in the clinic (American Society of Anesthesiologists, 2017). Research has indicated that in contrast to extended fasting, consuming water 2 h before anesthesia leads to decreased stomach contents and a rise in pH, thereby lowering the likelihood of intense pulmonary aspiration (Callaghan et al., 2016). Wang et al. (2019) verified that orally consuming carbohydrates 2 h before surgery does not increase reflux risk. Our research revealed that compared with the control group, there was no evidence that the incidence of aspiration, gastric reflux, the retention of gastric food or fluid in the study group had increased significantly. These findings indicate that reducing the duration of fasting and no drinking is relatively safe and practical. We also observed that the control group had a greater propensity for low blood pressure than the study group did, echoing findings from Oguzhan’s research (Yeniay et al., 2019).

Good visual field clarity ensures the accuracy of gastroscopy (Manfredi et al., 2023). There is a certain relationship between visual field clarity and gastric emptying (Burke, Harkins & Arumugasamy, 2024). Excessive stomach contents not only prolong the time of gastroscopy and increase the pain of the examinee, but also affect the accuracy of gastroscopy diagnosis. A study revealed that the gastric emptying ability of older patients was significantly slower than that of young patients when only liquid food was consumed, but there was no significant difference when solid food was consumed (Kao et al., 1994). On this basis, we orally administered 250 ml of liquid food and water before the painless gastroscopy instead of the maximum volume of 400 ml recommended by the ASA (Joshi et al., 2023). Moreover, the practice guidelines of the Indian Society of Anaesthesiologists state that the gastric emptying time of opaque liquid food is 2–4 h, while that of water is less than 2 h (Dongare et al., 2020). Therefore, we opted for the study group to take 250 ml of milk or rice porridge 4 h before painless gastroscopy and 250 ml of water 2 h before painless gastroscopy. Our results show that this intervention does not affect the clarity of gastric mucosal vision, and the fasting scheme adopted in this study is feasible for painless gastroscopy in older patients.

Furthermore, the results of this study revealed that the overall satisfaction of the study group markedly surpassed that of the control group, possibly because of the reduced duration of abstinence from water and food consumption before surgery, which decreased insulin resistance rates and mitigated stress reactions (Xu et al., 2017).

There are several limitations in this study that warrant mention. First, in this study, the degree of comfort indicators such as thirst, hunger, dizziness and fatigue are subjective indicators, lacking objective indicators. Second, our study was focused on complications during gastroscopy, and an in-depth study was not conducted on complications and resuscitation time during anesthesia, but this did not affect the conclusions of this article. Third, as the main observation index of this study was the comfort of older patients before painless gastroscopy, we did not perform statistical analysis on the painless gastroscopy examination time and complications after gastroscopy. Fourth, fasting has a certain effect on blood sugar, but considering that blood sugar testing is an invasive operation and can negatively affect older patients, this study did not test the blood sugar levels of older patients before and after painless gastroscopy. And ratings by a single endoscopist were also a limitation. Fifth, it must be noted that the primary patient-centered comfort outcomes emphasized in this article (thirst, hunger, dizziness, fatigue) were not explicitly listed as the primary outcome in the initial clinical trial registry entry, which focused on safety endpoints. This was an oversight in the registration process that we regret. Future studies will ensure perfect alignment between registered and reported primary outcomes from the outset. Last, this study lacks power to exclude a small increased risk of rare, serious complications (*e.g*., aspiration pneumonia), precluding definitive safety equivalence claims. A definitive assessment of safety would require a much larger, multi-center trial specifically powered for these critical endpoints.

## Conclusions

In summary, the ingestion of 250 ml of opaque liquid food 4 h prior to painless gastroscopy, followed by the consumption of 250 ml of water 2 h before the procedure, significantly increased the comfort and satisfaction of older patients. We observed no statistically significant increase in clinically identified gastric reflux, aspiration, or impaired endoscopic field clarity; however, the incidence of such adverse events was low, and the trial was not powered to exclude small increases in rare but serious complications. Larger studies would be required to definitively establish safety equivalence.

## Supplemental Information

10.7717/peerj.20929/supp-1 Supplemental Information 1 Raw data.

10.7717/peerj.20929/supp-2 Supplemental Information 2 CONSORT 2010 Checklist.

10.7717/peerj.20929/supp-3 Supplemental Information 3 Detailed registration information of Chinese clinical trial registration center: ChiCTR2300072760.

10.7717/peerj.20929/supp-4 Supplemental Information 4 Clinical trial study protocol.

## Additional Information and Declarations

### Data Availability

The following information was supplied regarding data availability:

The raw measurements are available in the Supplemental File.

### Clinical Trial Registration

The following information was supplied regarding Clinical Trial registration:

ChiCTR2300072760.
