# Supplementary material for: Impact of reducing the duration of fasting and no drinking on the experiences of older patients receiving painless gastroscopy: a randomized controlled trial
Source: PeerJ. 2026 Mar 11;14:e20929. doi: 10.7717/peerj.20929 (PMC12988733; doi:10.7717/peerj.20929)
Supplement: Supplemental Information 3 [file peerj-14-20929-s003.docx]

缩短禁食水时间对无痛胃镜老年受检者的就医体验影响研究

[下载XML文档](https://www.chictr.org.cn/bin/chictr/DownloadXml?path=ROg1XEiLWyYjY8jhKU2kOcMHGjBjbWewnzvsmEc5RLnZEZD9+heLNhMdmuLK1eGpQfLN8A/hnS+EgNSZYuMY4DSA4ikvDoOS1UKNGyZAxx/JYjlve3J1LTSSU/pNuU29/NRInJX2WyvJUte//Y8O/0WhuXAZpdShGJKxhupiO90=)

| 注册号：  Registration number： | ChiCTR2300072760 | | |
| --- | --- | --- | --- |
| 最近更新日期：  Date of Last Refreshed on： | 2023-08-21 | | |
| 注册时间：  Date of Registration： | 2023-06-25 | | |
| 注册号状态： | 预注册 | | |
| Registration Status： | Prospective registration | | |
| 注册题目： | 缩短禁食水时间对无痛胃镜老年受检者的就医体验影响研究 | | |
| Public title： | Research on shortening the time of fasting and water forbidden of painless gastroscopy elderly patients | | |
| 注册题目简写： |  | | |
| English Acronym： |  | | |
| 研究课题的正式科学名称： | 缩短禁食水时间对无痛胃镜老年受检者的就医体验影响研究 | | |
| Scientific title： | Research on shortening the time of fasting and water forbidden of painless gastroscopy elderly patients | | |
| 研究课题代号(代码)：  Study subject ID： |  | | |
| 在二级注册机构或其它机构的注册号：  The registration number of the Partner Registry or other register： |  | | |
| 申请注册联系人： | 兰勇 | 研究负责人： | 兰勇 |
| Applicant： | Lan Yong | Study leader： | Lan Yong |
| 申请注册联系人电话：  Applicant telephone： | +86 134 5873 9825 | 研究负责人电话：  Study leader's telephone： | +86 134 5873 9825 |
| 申请注册联系人传真 ：  Applicant Fax： |  | 研究负责人传真：  Study leader's fax： |  |
| 申请注册联系人电子邮件：  Applicant E-mail： | 1025213332@qq.com | 研究负责人电子邮件：  Study leader's E-mail： | 1025213332@qq.com |
| 申请单位网址(自愿提供)：  Applicant website(voluntary supply)： |  | 研究负责人网址(自愿提供)：  Study leader's website(voluntary supply)： |  |
| 申请注册联系人通讯地址： | 四川省泸州市龙马潭区春晖路182号 | 研究负责人通讯地址： | 四川省泸州市龙马潭区春晖路182号 |
| Applicant address： | 182 Chunhui Road, Longmatan District, Luzhou, Sichuan | Study leader's address： | 182 Chunhui Road, Longmatan District, Luzhou, Sichuan |
| 申请注册联系人邮政编码：  Applicant postcode： |  | 研究负责人邮政编码：  Study leader's postcode： |  |
| 申请人所在单位： | 西南医科大学附属中医医院 | | |
| Applicant's institution： | Southwest Medical University Hospital of Traditional Chinese Medicine | | |
| 研究负责人所在单位： | 西南医科大学附属中医医院 | | |
| Affiliation of the Leader： | Southwest Medical University Hospital of Traditional Chinese Medicine | | |

| 是否获伦理委员会批准： | | 是 | | |
| --- | --- | --- | --- | --- |
| Approved by ethic committee： | | Yes | | |
| 伦理委员会批件文号：  Approved No. of ethic committee： | | BY2023010 | 伦理委员会批件附件：  Approved file of Ethical Committee： | 查看附件View |
| 批准本研究的伦理委员会名称： | | 西南医科大学附属中医医院医学伦理审查委员会 | | |
| Name of the ethic committee： | | Medical Ethics Review Committee of the Affiliated Hospital of Traditional Chinese Medicine of Southwest Medical University | | |
| 伦理委员会批准日期：  Date of approved by ethic committee： | | 2023-05-12 | | |
| 伦理委员会联系人： | | 贾强 | | |
| Contact Name of the ethic committee： | | Jia Qiang | | |
| 伦理委员会联系地址： | | 四川省泸州市龙马潭区春晖路182号 | | |
| Contact Address of the ethic committee： | | 182 Chunhui Road, Longmatan District, Luzhou, Sichuan | | |
| 伦理委员会联系人电话：  Contact phone of the ethic committee： | | +86 830 251 6312 | 伦理委员会联系人邮箱：  Contact email of the ethic committee： |  |
| 研究实施负责（组长）单位： | 西南医科大学附属中医医院 | | | |
| Primary sponsor： | Southwest Medical University Hospital of Traditional Chinese Medicine | | | |
| 研究实施负责（组长）单位地址： | 四川省泸州市龙马潭区春晖路182号 | | | |
| Primary sponsor's address： | 182 Chunhui Road, Longmatan District, Luzhou, Sichuan | | | |
| 试验主办单位(项目批准或申办者)：  Secondary sponsor： | \| 国家： \| 中国 \| 省(直辖市)： \| 四川 \| 市(区县)： \| 泸州 \| \| --- \| --- \| --- \| --- \| --- \| --- \| \| Country： \| China \| Province： \| Sichuan \| City： \| Luzhou \| \| 单位(医院)： \| 西南医科大学附属中医医院 \| 具体地址： \| 四川省泸州市龙马潭区春晖路182号 \| \|  \| \| Institution hospital： \| Southwest Medical University Hospital of Traditional Chinese Medicine \| Address： \| 182 Chunhui Road, Longmatan District, Luzhou, Sichuan \| \|  \| | | | |
| 经费或物资来源： | 无 | | | |
| Source(s) of funding： | None | | | |
| 研究疾病： | 上消化道疾病 | | | |
| Target disease： | Upper Gastrointestinal Diseases | | | |
| 研究疾病代码： |  | | | |
| Target disease code： |  | | | |
| 研究类型： | 干预性研究 | | | |
| Study type： | Interventional study | | | |
| 研究所处阶段： | 治疗新技术临床试验 | | | |
| Study phase： | New Treatment Measure Clinical Study | | | |
| 研究设计： | 随机平行对照 | | | |
| Study design： | Parallel | | | |
| 研究目的： | 通过缩短无痛胃镜检查前禁饮禁食时间，以探讨其对老年患者就医体验的影响。 | | | |
| Objectives of Study： | By reducing the duration of fasting before painless gastroscopy in order to explore its impact on the experience of elderly patients. | | | |
| 药物成份或治疗方案详述： |  | | | |
| Description for medicine or protocol of treatment in detail： |  | | | |
| 纳入标准： | 1.年龄≥65岁； 2.ASA Ⅰ或Ⅱ级； 3.无相关药物禁忌症。 | | | |
| Inclusion criteria | 1. Age >= 65 years; 2. ASA class I or II; 3. No contraindications to relevant medication. | | | |
| 排除标准： | 1.严重心血管系统疾病及呼吸道病变； 2.肝功能障碍 (Child-Pugh C级以上) 、急性上消化道出血伴休克、胃肠道梗阻； 3.有镇静/麻醉药物过敏及其他严重麻醉风险者。 | | | |
| Exclusion criteria： | 1. Severe cardiovascular system disease and respiratory pathology; 2. Liver dysfunction (Child-Pugh class C or above), acute upper gastrointestinal bleeding with shock, gastrointestinal obstruction; 3. Hypersensitivity to sedative/anaesthetic drugs and other serious anaesthetic risks. | | | |

| 研究实施时间：  Study execute time： | 从 From 2023-07-01至 To 2023-12-01 | 征募观察对象时间：  Recruiting time： | 从 From 2023-07-01 至 To 2023-12-01 |
| --- | --- | --- | --- |

| 干预措施：  Interventions： | \| 组别： \| 对照组 \| 样本量： \| 450 \| \| --- \| --- \| --- \| --- \| \| Group： \| Control group \| Sample size： \| \| 干预措施： \| 胃镜检查前常规禁食6～8h、禁水4h \| 干预措施代码： \|  \| \| Intervention： \| Routine fasting for 6-8h, water fasting for 4h before gastroscopy \| Intervention code： \|  \| 组别： \| 研究组 \| 样本量： \| 450 \| \| --- \| --- \| --- \| --- \| \| Group： \| Research Group \| Sample size： \| \| 干预措施： \| 胃镜检查前禁食4h（进食牛奶或米粥250ml），禁水2h \| 干预措施代码： \|  \| \| Intervention： \| 4h fasting (250ml of milk or rice porridge) and 2h water fasting before gastroscopy \| Intervention code： \| |
| --- | --- | --- | --- | --- | --- | --- | --- | --- | --- | --- | --- | --- | --- | --- | --- | --- | --- | --- | --- | --- | --- | --- | --- | --- | --- | --- | --- | --- | --- |
| 研究实施地点：  Countries of recruitment and research settings： | \| 国家： \| 中国 \| 省(直辖市)： \| 四川 \| 市(区县)： \| 泸州 \| \| --- \| --- \| --- \| --- \| --- \| --- \| \| Country： \| China \| Province： \| Sichuan \| City： \| Luzhou \| \| 单位(医院)： \| 西南医科大学附属中医医院 \| 单位级别： \| 三甲 \| \| \| \| Institution hospital： \| Southwest Medical University Hospital of Traditional Chinese Medicine \| Level of the institution： \| Tertiary A \| \| \| |

| 测量指标：  Outcomes： | \| 指标中文名： \| 麻醉风险：有无胃内液体反流、胃食物潴留、胃液体潴留、有无误吸等； \| 指标类型： \| 主要指标 \| \| --- \| --- \| --- \| --- \| \| Outcome： \| Risks of anaesthesia: presence of gastric fluid reflux, gastric food retention, gastric fluid retention, presence of aspiration by mistake, etc \| Type： \| Primary indicator \| \| 测量时间点： \|  \| 测量方法： \|  \| \| Measure time point of outcome： \|  \| Measure method： \|  \|  \| 指标中文名： \| 患者检查前舒适度：口渴、饥饿、头晕、乏力等不适发生率 \| 指标类型： \| 次要指标 \| \| --- \| --- \| --- \| --- \| \| Outcome： \| Patient comfort before examination: incidence of discomfort such as thirst, hunger, dizziness and fatigue \| Type： \| Secondary indicator \| \| 测量时间点： \|  \| 测量方法： \|  \| \| Measure time point of outcome： \|  \| Measure method： \|  \|  \| 指标中文名： \| 视野清晰度 \| 指标类型： \| 次要指标 \| \| --- \| --- \| --- \| --- \| \| Outcome： \| Field of view clarity \| Type： \| Secondary indicator \| \| 测量时间点： \|  \| 测量方法： \|  \| \| Measure time point of outcome： \|  \| Measure method： \|  \|  \| 指标中文名： \| 患者总体满意度 \| 指标类型： \| 次要指标 \| \| --- \| --- \| --- \| --- \| \| Outcome： \| Overall patient satisfaction \| Type： \| Secondary indicator \| \| 测量时间点： \|  \| 测量方法： \|  \| \| Measure time point of outcome： \|  \| Measure method： \|  \| |
| --- | --- | --- | --- | --- | --- | --- | --- | --- | --- | --- | --- | --- | --- | --- | --- | --- | --- | --- | --- | --- | --- | --- | --- | --- | --- | --- | --- | --- | --- | --- | --- | --- | --- | --- | --- | --- | --- | --- | --- | --- | --- | --- | --- | --- | --- | --- | --- | --- | --- | --- | --- | --- | --- | --- | --- | --- | --- | --- | --- | --- | --- | --- | --- | --- | --- |
| 采集人体标本：  Collecting sample(s) from participants： | \| 标本中文名： \| 无 \| 组织： \|  \| \| --- \| --- \| --- \| --- \| \| Sample Name： \| None \| Tissue： \|  \| \| 人体标本去向 \| 其它 \| 说明 \|  \| \| Fate of sample： \| 0thers \| Note： \|  \| |

| 征募研究对象情况：  Recruiting status： | 正在进行  Recruiting | 年龄范围：  Participant age： | \| 最小 Min age \| 65 \| 岁 years \| \| --- \| --- \| --- \| \| 最大 Max age \| 100 \| 岁 years \| |
| --- | --- | --- | --- | --- | --- | --- | --- | --- | --- |
| 性别： | 男女均可 | Gender： | Both |
| 随机方法（请说明由何人用什么方法产生随机序列）： | 采用随机数字表法，通过不透光的密封信封将纳入的患者，随机分为研究组和对照组。 | | |
| Randomization Procedure (please state who generates the random number sequence and by what method)： | The random number table method was used to randomise the included patients, via opaque sealed envelopes, into a study group and a control group. | | |
| 是否公开试验完成后的统计结果:  Calculated Results after the Study Completed public access: | 公开/Public | | |

| 盲法： | 单盲，评估检查的研究者对患者的组别不知情。 |
| --- | --- |
| Blinding： | Single blind, the researcher conducting the evaluation examination is unaware of the patient's group. |
| 试验完成后的统计结果（上传文件）： | [点击下载](https://www.chictr.org.cn/bin/chictr/DownloadTest?path=) |
| Calculated Results after the Study Completed(upload file)： | [download](https://www.chictr.org.cn/bin/chictr/DownloadTest?path=) |
| 是否共享原始数据：  IPD sharing | 否No |
| 共享原始数据的方式（说明：请填入公开原始数据日期和方式，如采用网络平台，需填该网络平台名称和网址）： | 无 |
| The way of sharing IPD”(include metadata and protocol, If use web-based public database, please provide the url)： | None |
| 数据采集和管理（说明：数据采集和管理由两部分组成，一为病例记录表(Case Record Form, CRF)，二为电子采集和管理系统(Electronic Data Capture, EDC)，如ResMan即为一种基于互联网的EDC： | 病例记录表 |
| Data collection and Management (A standard data collection and management system include a CRF and an electronic data capture： | CRF |
| 数据与安全监察委员会：  Data and Safety Monitoring Committee： | 暂未确定/Not yet |
