# Supplementary material for: Impact of reducing the duration of fasting and no drinking on the experiences of older patients receiving painless gastroscopy: a randomized controlled trial
Source: PeerJ. 2026 Mar 11;14:e20929. doi: 10.7717/peerj.20929 (PMC12988733; doi:10.7717/peerj.20929)
Supplement: Supplemental Information 4 [file peerj-14-20929-s004.docx]

附件6

# “缩短禁食水时间对无痛胃镜老年受检者的就医体验影响研究”研究方案

Study on the impact of reducing the duration of fasting and no drinking on the experience of older patients receiving painless gastroscopy

**研究者：兰勇**

**Investigator: Yong Lan**

**研究单位：西南医科大学附属中医医院**

**Study Unit: Traditional Chinese Medicine Hospital affiliated to Southwest Medical University.**

**参加单位：/**

**Participating units: /**

**研究周期：3月**

**Research period: 3 months.**

**缩略语对照**

| 缩略词 | 中文名称 |
| --- | --- |
| ASA | 美国麻醉医师协会 |
| ERAS | 加速康复外科 |
| AE | 不良事件 |

**摘 要Abstract**

| 题目 Title | 缩短禁食水时间对无痛胃镜老年受检者的就医体验影响研究  Study on the impact of reducing the duration of fasting and no drinking on the experience of older patients receiving painless gastroscopy |
| --- | --- |
| 牵头单位  Lead unit | 西南医科大学附属中医医院Traditional Chinese Medicine Hospital affiliated to Southwest Medical University. |
| 参与单位Participating units | / |
| 主要研究者  Main investigator | 陈玥熙、田佳鑫、江霞、李时超、孙欣 Yuexi Chen, Jiaxin Tian, Xia Jiang, Shichao Li, Xin Sun |
| 计划受试者数  Number of planned subjects | 900例 900 cases |
| 研究目的Study purposes | 通过缩短无痛胃镜检查前禁饮禁食时间，以探讨其对老年患者就医体验的影响。By shortening the time of no drinking and fasting before painless gastroscopy, this study discusses its influence on the medical experience of older patients. |
| 研究方法Sudy method | 采用随机、双盲、对照临床试验Randomized, double-blind and controlled clinical trials were used. |
| 主要入选标准   Main selection criteria | 1. 年龄≥65岁；age ≥65 years; 2. ASA分级：Ⅰ或Ⅱ级； classified as ASA class I or II; 3. 无相关药物禁忌症。presented no significant drug contraindications. |
| 主要排除标准  Main exclusion criteria | 1. 严重心血管系统疾病及呼吸道病变； 2. 肝功能障碍 (Child-Pugh C级以上) 、急性上消化道出血伴休克、胃肠道梗阻； 3. 有镇静/麻醉药物过敏及其他严重麻醉风险者。 4. critical cardiovascular and respiratory conditions; 5. liver dysfunction (Child-Pugh class C or above), sudden upper gastrointestinal hemorrhage accompanied by shock, blockage in the gastrointestinal tract; 6. allergic reactions to sedatives/anesthetics, along with other grave anesthesia hazards. |
| 干预措施  Intervention measure | 通过随机数字表法，通过不透光的密封信封将纳入的患者，随机分为研究组和对照组。研究组检查前禁食4h（进食牛奶或米粥250ml），禁水2h；对照组则常规禁食6～8h、禁水4h。检查前一天由专职护士向患者解释禁饮禁食的目的，增强患者的配合度，并于检查当日记录患者禁饮禁食时间及禁食性质（固体、液体）；选取口渴、饥饿、头晕、乏力四个条目作为舒适度指标，由专职护士于麻醉诱导前对两组患者进行评估。胃镜检查结束后，通过问卷形式记录受检者对此次胃镜检查满意度，并由操作的内镜医师评价胃准备情况、评估视野清晰度。The patients were randomly divided into study group and control group by random number table method and sealed envelope. The study group fasted for 4 hours before examination (eating 250ml of milk or rice porridge) and banned water for 2 hours; The control group was routinely fasted for 6 ~ 8 hours and no drinking for 4 hours. The day before the examination, the full-time nurse explained the purpose of drinking and fasting to the patients to enhance their cooperation, and recorded the time of drinking and fasting and the nature of fasting (solid and liquid) on the day of the examination; Four items, thirst, hunger, dizziness and fatigue, were selected as comfort indexes, and the full-time nurses evaluated the two groups of patients before anesthesia induction. After the gastroscopy, the satisfaction of the subjects with the gastroscopy was recorded by questionnaire, and the operating endoscopist evaluated the gastric preparation and the visual field clarity. |
| 主要观察指标  Main outcome measures | 观察指标：（1）患者检查前舒适度：口渴、饥饿、头晕、乏力等不适发生率；（2）无痛胃镜检查中安全性：胃镜检查完成后由检查医师填写和评价胃镜检查期间的安全性，包括有无胃内液体反流、胃食物潴留、胃液体潴留、有无误吸等；（3）视野清晰度：由检查医师根据检查时黏膜可视清晰度进行判断，以李克特量表5分法进行评估。胃镜下黏膜观察受限程度：不受限（0分）、轻度受限（1-3分）、中度受限（4-6分）、重度受限（7-9分）、无法观察（10分）；（4）患者总体满意度，以0-100分评分。  Observation indicators: (1) Comfort before examination: incidence of discomfort such as thirst, hunger, dizziness and fatigue; (2) Safety in painless gastroscopy: After the gastroscopy is completed, the examining physician should fill in and evaluate the safety during gastroscopy, including whether there is gastric fluid reflux, gastric food retention, gastric fluid retention, and whether there is any correct aspiration. (3) Visibility: The examining physician will judge according to the visual clarity of mucosa during examination, and evaluate it with 5-point Likert scale. The limited degree of mucosal observation under gastroscope: unrestricted (0), slightly limited (1-3), moderately limited (4-6), severely limited (7-9), unable to observe (10); (4) Patients' overall satisfaction, with a score of 0-100. |
| 安全性评价   Safety evaluation | 胃镜检查完成后由检查医师填写和评价胃镜检查期间的安全性，包括有无胃内液体反流、胃食物潴留、胃液体潴留、有无误吸等；对不良事件参照2017年美国常⻅不良事件评价标准（CTCAE）5.0 进行分级  1级：轻度症状；无需治疗；  2级：中度症状；需要干预治疗；  3级：需要输血治疗；需要有创干 预治疗或者住院治疗;  4级：危及生命；需要紧急治疗  5级：死亡  After the completion of gastroscopy, the examining physician shall fill in and evaluate the safety during gastroscopy, including whether there is gastric liquid reflux, gastric food retention, gastric liquid retention, and correct aspiration; Adverse events were classified according to the 2017 American Common Adverse Event Evaluation Standard (CTCAE)5.0.  Grade 1: mild symptoms; No need for treatment;  Grade 2: moderate symptoms; Need intervention treatment;  Grade 3: need blood transfusion treatment; Need pre-treatment or hospitalization for invasive trunk;  Level 4: life-threatening; Need urgent treatment  Level 5: Death |
| 研究时间Study time | 2023.3-2023.6 |
| 统计方法 Statistical method | 采用 SPSS23.0统计软件进行统计学分析，正态分布计量资料以均数±标准差( ‾x±s)表示，正态分布采用t 检验。非正态分布采用非参数秩和检验，计数资料采用χ^2^检验，P＜0.05 为差异具有统计学意义。  Statistical analysis was carried out by SPSS23.0 statistical software. The measurement data of normal distribution were expressed by the mean standard deviation (‾x±s), and the normal distribution was tested by t test. The nonparametric rank sum test was used for the non-normal distribution, and the 2 test was used for the counting data, and the difference was statistically significant (P < 0.05). |

**目 录**

1.立项依据

1.1立项的必要性及国内外研究现状

(一)立项必要性

现代医疗水平的提升使得人类寿命显著延长，据WHO报道，全球预期寿命已增长至73.3岁，在理想世界中，寿命越长则说明身体健康时间越长，但自2000年以来，情况却恰恰相反,寿命的延长伴随而来的是健康状况的每况愈下^[1]^。统计也显示，我国即将进入世界卫生组织所定义的深度老龄化社会^[2]^。随着年龄的增长，老年人各个器官功能衰退，免疫力下降，多种疾病随之袭来，其中消化系统疾病的患病率同样也会增加。胃镜检查正是上消化系统疾病诊治的重要手段之一，舒适化医疗的发展使得无痛胃镜成为患者们的首选。术前禁饮禁食是无痛胃镜检查的必要条件，严格的禁饮禁食可以减少反流和误吸诱发的致命性吸入性肺炎及其他严重并发症^[3-4]^。然而随着增强术后恢复（ERAS）的概念受到广泛关注^[5-6]^，研究表明，术前过度禁食会导致营养不良，这种营养不良是造成住院时间延长、再入院率和死亡率增加的原因^[7]^。经过多年的临床实践，传统的禁饮禁食概念已经受到了外科医生和麻醉师的质疑。

(二)国内外研究现状

无痛胃镜是利用一种或多种药物，保证患者在无痛、安全、适宜的浅睡眠情况下进行胃部内镜检查及治疗的技术^[8]^。因其创伤小、恢复快等优点，目前已在国内各大医院广泛应用^[9]^。为了避免患者在麻醉期间出现吸入性肺炎等严重并发症，无痛内镜操作共识中明确指出，行无痛胃镜检查前，患者需常规禁食 6-8h，禁饮 4h^[10]^。然而在实际临床操作中，绝大多数无痛胃镜检查患者的禁饮禁食时间远超于此，更有甚者可以长达至16小时^[11]^。长时间的禁饮禁食会给患者的心理和生理带来双重压力^[12]^。患者易出现口渴、饥饿、烦躁、焦虑、电解质紊乱及代谢功能异常等不良反应，甚至增加麻醉后反流的风险^[13-14]^，尤其在老年患者这一特殊群体中体现的更为明显。由于老年患者组织器官功能的衰退，常常会合并多种年龄相关性慢性疾病且长期口服多种治疗药物^[15]^，大大增加了麻醉风险。加之长时间的禁饮禁食，常导致老年患者在准备期间就出现头晕、头痛、恶心等一系列症状^[16]^，进而在操作过程中引发低血压、心律失常等并发症。这些由于过长的禁食禁饮时间所致的不良反应，大大降低了老年患者的身心舒适度，影响了其就医体验。此前，美国麻醉师协会（ASA）建议患者可在术前2小时口服适量的透明液体^[17]^，但对于老年患者来说，仍缺乏安全性和可操作性分析。

(三) 研究意义

综上，缩短无痛胃镜检查前禁食禁水时间符合加速康复外科理念，但对于进行无痛胃镜检查的老年患者来说，仍缺乏安全性和可操作性分析。因此，本研究将以行无痛胃镜的老年人为研究对象，通过随机、对照、单中心的前瞻性临床试验探讨缩短无痛胃镜检查前禁饮禁食时间对老年患者就医体验的影响，以提高老年患者的舒适度及满意度，在临床广泛推广。

1.2主要参考文献

[1]World Health Organization. Geneva: World Health Organization; 2021. World health statistics 2021: monitoring health for the SDGs, sustainable development goals

[2]张耀军,齐婧含.“十四五”时期中国人口发展的重大问题[J].哈尔滨工业大学学报(社会科学版),2022,24(02):144-153.

[3]Andersson Hanna,Schmitz Achim,Frykholm Peter,Preoperative fasting guidelines in pediatric anesthesia: are we ready for a change?[J] .Curr Opin Anaesthesiol, 2018, 31: 342-348.

[4]Thomas Mark,Morrison Christa,Newton Richard et al. Consensus statement on clear fluids fasting for elective pediatric general anesthesia.[J] .Paediatr Anaesth, 2018, 28: 411-414.

[5]Parks Lisa,Routt Meghan,De Villiers Allison,Enhanced Recovery After Surgery.[J] .J Adv Pract Oncol, 2018, 9: 511-519.

[6]Brown Daran,Xhaja Anisa,Nursing Perspectives on Enhanced Recovery After Surgery.[J] .Surg Clin North Am, 2018, 98: 1211-1221.

[7]De Jonghe B,Fajardy A,Mérian-Brosse L et al. Reducing pre-operative fasting while preserving operating room scheduling flexibility: feasibility and impact on patient discomfort.[J] .Acta Anaesthesiol Scand, 2016, 60: 1222-9.

[8]Ladas SD, Satake Y, Mostafa I, et al. Sedation practices for gastrointestinal endoscopy in Europe, North America, Asia, Africa and Australia[J]. Digestion, 2010, 82(2): 74-76.

[9]张修礼,唐平,孔金艳,路新卿,杨云生.国内无痛胃肠镜开展近况调查[J].中华消化内镜杂志,2012(06):316-318.

[10]中国医师协会消化医师分会.无痛消化内镜操作共识[J].中国实用内科杂志，2010，30（7）：605-607.

[11]王书智,付立,吴军,时之梅,王淑萍,陆蕊,叶志霞.胃镜检查前禁水时间对检查清晰度和患者安全性的影响[J].解放军护理杂志,2015,32(12):50-52.

[12]Lua Bee Chen,Md Hashim Mohd Nizam,Wong Mung Seong et al. Efficacy and safety of pre-gastroscopy commercial carbohydrate-rich whey protein beverage vs. plain water: a randomised controlled trial.[J] .Sci Rep, 2022, 12: 17355.

[13]Ducey Ariel,Nikoo Shoghi,Formats of responsibility: elective surgery in the era of evidence-based medicine.[J] .Sociol Health Illn, 2018, 40: 494-507.

[14]Napolitano Michael A,Sparks Andrew D,Randall J Alex et al. Elective surgery for diverticular disease in U.S. veterans: A VASQIP study of national trends and outcomes from 2004 to 2018.[J] .Am J Surg, 2021, 221: 1042-1049.

[15]Rieckert Anja,Trampisch Ulrike S,Klaaßen-Mielke Renate et al. Polypharmacy in older patients with chronic diseases: a cross-sectional analysis of factors associated with excessive polypharmacy.[J] .BMC Fam Pract, 2018, 19: 113.

[16]Leslie K, Tay T, Neo E. Intravenous fluid to prevent hypotension in patients undergoing elective colonoscopy [J]. Anaesth Intensive Care, 2006, 34(3): 316-321.

[17]Practice Guidelines for Preoperative Fasting and the Use of Pharmacologic Agents to Reduce the Risk of Pulmonary Aspiration: Application to Healthy Patients Undergoing Elective Procedures: An Updated Report by the American Society of Anesthesiologists Task Force on Preoperative Fasting and the Use of Pharmacologic Agents to Reduce the Risk of Pulmonary Aspiration.[J] .Anesthesiology, 2017, 126: 376-393.

**2.研究设计**

2.1研究目的

通过缩短无痛胃镜检查前禁饮禁食时间，以探讨其对老年患者就医体验的影响。

2.2研究方法

单中心、随机、对照、双盲试验。

2.3研究内容

选取2023年3月至2023年6月在西南医科大学附属中医医院行无痛胃镜检查的老年患者，采用随机数字表法，通过不透光的密封信封将纳入的患者，随机分为研究组和对照组。研究组检查前禁食4h（进食牛奶或米粥250ml），禁水2h；对照组则常规禁食6～8h、禁水4h。检查前一天由专职护士向患者解释禁饮禁食的目的，增强患者的配合度，并于检查当日记录患者禁饮禁食时间及禁食性质（固体、液体）；选取口渴、饥饿、头晕、乏力四个条目作为舒适度指标，由专职护士于麻醉诱导前对两组患者进行评估。胃镜检查结束后，通过问卷形式记录受检者对此次胃镜检查满意度，并由操作的内镜医师评价胃准备情况、评估视野清晰度。

2.4技术关键

无痛胃镜的操作作为本次研究的重要手段。在操作过程中，不同的医师最终可能会有不同的临床效果，研究者会遵循统一的操作规范，并且研究前都经过研究主持者的手术操作培训，避免了操作医师因素对试验的影响。通过前瞻性研究，客观探讨缩短禁食水时间对无痛胃镜老年受检者的就医体验影响。

2.5技术路线

**
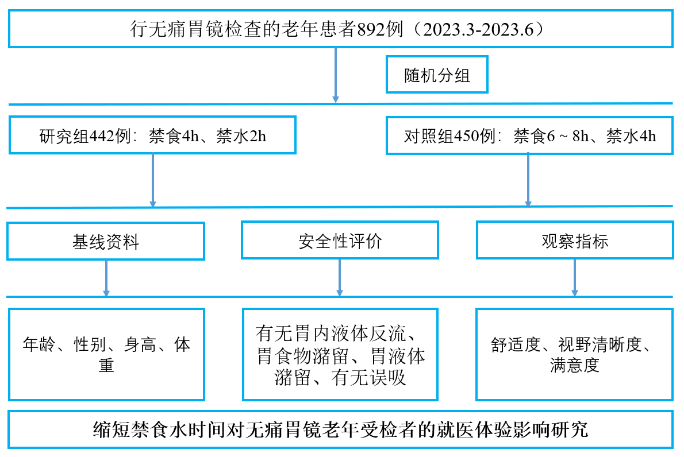
**

2.6研究方案

2.6.1样本量及分组

选取2023年3月至2023年6月在西南医科大学附属中医医院行无痛胃镜检查的所有老年患者。按照1：1的比例分为研究组和对照组纳入例数，参考文献报道，取δ=-1.5，α为 0.05，采用双侧检验，预估中途脱落率为5%，根据非劣效试验样本量计算公式计算出每组样本450例，即研究组450例，对照组450例，总纳入病例为900例。研究组检查前禁食4h（进食牛奶或米粥250ml），禁水2h；对照组则常规禁食6～8h、禁水4h。

2.6.2纳入标准

1. 年龄≥65岁；
2. ASA Ⅰ或Ⅱ级；
3. 无相关药物禁忌症。

2.6.3排除标准

1. 严重心血管系统疾病及呼吸道病变；
2. 肝功能障碍 (Child-Pugh C级以上) 、急性上消化道出血伴休克、胃肠道梗阻；
3. 有镇静/麻醉药物过敏及其他严重麻醉风险者。

2.6.4观察指标

2.6.4.1一般资料观察

- 1. 人口学资料：包括年龄、性别、身高、体重；

2.6.4.2安全性指标

胃镜检查完成后由检查医师填写和评价胃镜检查期间的安全性，包括有无胃内液体反流、胃食物潴留、胃液体潴留、有无误吸等；

2.6.4.3观察指标

1. 患者检查前舒适度：口渴、饥饿、头晕、乏力等不适发生率；
2. 视野清晰度：由检查医师根据检查时黏膜可视清晰度进行判断，以李克特量表5分法进行评估。胃镜下黏膜观察受限程度：不受限（0分）、轻度受限（1-3分）、中度受限（4-6分）、重度受限（7-9分）、无法观察（10分）；
3. 患者总体满意度，以0-100分评分。

**2.5数据管理与统计分析**

采用EXCEL汇总数据后，采用 SPSS23.0统计软件进行统计学分析，正态分布计量资料以均数±标准差( ‾x±s)表示，正态分布采用t 检验。非正态分布采用非参数秩和检验，计数资料采用χ^2^检验，P＜0.05 为差异具有统计学意义。

**2.6伦理学要求**

临床试验必须遵循赫尔辛基宣言和我国有关临床试验研究规范、法规进行。在试验开始之前，试验方案应由伦理委员会批准后方可实施临床试验。

每一位患者入选本研究前，研究医师有责任以书面文字形式，向其或其指定代表人完整、全面地介绍本研究的目的、程序和可能的风险，应让患者知道他们有权随时退出本研究。入选前必须给每位患者或其亲属一份书面知情同意书，研究医师有责任让每位患者在进入研究之前获得知情同意，知情同意书应作为临床试验文档保留备查。

研究者负责受试者的医疗，作出与临床试验相关的医疗决定，保证受试者在试验期间出现不良事件时得到适当的治疗。研究者对研究所发生的严重不良事件，应采取必要的措施以保证受试者的安全和权益，并及时向伦理委员会报告。

试验期间如果发生与试验有关的不良事件，受试者将得到免费的医疗。治疗中如病情变化，患者或家属应及时和研究者联系，研究者可根据自己的判断，决定是否进行其他处理。研究者应对试验相关的损害或死亡的受试者承担治疗的费用及相应的经济补偿。

在不违反保密原则和相关法规的情况下，伦理委员会和相关管理部门人员可以查阅受试者的原始医学记录，以核实临床试验的过程和数据。除法规允许外，受试者参加临床试验的相关记录应保密，不得公开。受试者的临床试验资料将保存在医院相关管理部门。如果发布试验结果，受试者的身份信息仍应保密。

**2.7质量保证**

本试验将遵循赫尔辛基宣言及相关法律法规开展。研究团队必须完整阅读研究方案并严格遵循其条例。

病例报告表每个未填的数据应给出足够的解释。改正数据应注明日期、签名及修改的正当理由，如果必要由修改者本人说明。研究者要保留所有有关本研究的记录（包括已经退出本研究的受试者的资料）。

主要研究者应召集所有主要研究者和参与者，对研究方案、流程、病例报告表广泛培训和讨论，并明确各研究者的职责。

**2.8研究进度**

| 2023-03-01 | 2023-03-31 | 完成临床试验的伦理审批、海报宣传；收集临床病例入组。 |
| --- | --- | --- |
| 2023-04-01 | 2023-06-30 | 继续入组前瞻性研究的病例；汇总、统计分析数据 |

**2.9预期结果**

将无痛胃镜检查前禁食6～8h、禁水4h精准调控为禁食4h、禁水2h，可以提高老年患者的舒适度及满意度，且对胃镜检查时胃黏膜的可视清晰度无影响，同时并不增加检查中胃液反流及误吸的风险，安全可行。

**3、研究基础**

**3.1与本课题有关的前期研究状况**

暂无。

**3.2实验设备、工作条件及技术力量**

腔镜中心是本院为满足大型中西医结合医院发展需求投入巨资建设的省级重点专科，腔镜中心建筑面积达1600多平方米，具有消化内镜、腹腔镜等诊疗技术。腔镜中心实现了一体化的管理，各功能布局分区合理，环境温馨、舒适。设施设备均达到国内外先进水平，现拥有医护人员20余名均具有过硬的、专业的诊疗能力和技术。中心长期开展无痛肠镜检查和治疗，年完成诊疗量20000余人次，并长期开展内镜下微创治疗：

**3.3近三年主持或主研的科研课题及成果**

3.2.1课题：

校级课题：肠炎灌肠保留灌肠对低位直肠癌预防性造瘘术后旷置性肠炎的疗效研究

3.2.2.既往发表文章

1. 余腾江,李五生,李时超,陈卫东,兰勇,杨向东,贾英田.13例直肠癌术后吻合口管状狭窄行内镜下支架置入治疗的效果分析[J].结直肠肛门外科,2022,28(06):603-606.
2. 兰勇,余腾江.中西医保留灌肠治疗旷置性肠炎的临床研究[J].医学食疗与健康,2022,20(09):29-32.
3. 兰勇,李五生.消化内镜在急性非静脉曲张性上消化道出血治疗中的应用价值[J].国际感染病学(电子版),2020,9(02):136.
